# Tissue Factor and Tissue Factor Pathway Inhibitor in Chronically Inflamed Gallbladder Mucosa

**DOI:** 10.1155/2014/403639

**Published:** 2014-02-27

**Authors:** Jacek Liczko, Tomasz Stawski, Małgorzata Żaba, Józef Kurek, Daniel Sabat, Grzegorz Wyrobiec, Dorota Domal-Kwiatkowska, Damian Dudek, Marek Kucharzewski, Krzysztof Helewski

**Affiliations:** ^1^Department of Histology and Embryology, Medical University of Silesia in Katowice, Jordana Street 19, 41-808 Zabrze, Poland; ^2^Municipal Hospital, Chełmońskiego Street 28, 43-600 Jaworzno, Poland; ^3^Department of Pathomorphology, Medical University of Silesia in Katowice, 3-go Maja Street 13-15, 41-800 Zabrze, Poland; ^4^Department of Biochemistry, Medical University of Silesia in Katowice, Jedności Street 8, 41-200 Sosnowiec, Poland; ^5^Department of Descriptive and Topographic Anatomy, Medical University of Silesia in Katowice, Jordana Street 19, 41-808 Zabrze, Poland

## Abstract

We characterised a tissue factor (TF) and tissue factor pathway inhibitor (TFPI) expression in relation to severity of inflammatory infiltration of the gallbladder mucosa in a chronic cholecystitis. We prospectively studied the gallbladder specimens obtained from 54 patients who had undergone cholecystectomy due to chronic calculous cholecystitis and 16 calculosis-free gallbladder specimens obtained from patients who underwent cholecystectomy due to the polyp/polyps as well as in cases of gallbladder injury. To assess TF and TFPI immunoreactivity by immunohistochemistry, the monoclonal anti-human TF and TFPI antibodies were used. The inflammatory infiltration of the gallbladder mucosa was reflected by the number of CD3 and CD68 positive cells. The expression of TF and TFPI differed significantly between the cholecystitis and the control group. Most capillary endothelial cells of the cholecystitis group presented weak expression for TFPI. The mean number of CD3 positive lymphocytes in the cholecystitis group was 18.6 ± 12.2, but the mean number of CD68 positive cells was 29.7 ± 13.9. In the control sections, it was 3.1 ± 1.9 and 8.8 ± 3.9, respectively (*P* < 0.001). The results of the current study suggest that the tissue procoagulant state found may be engaged in the etiopathogenesis of the cholecystitis.

## 1. Introduction

Chronic cholecystitis is characterised by chronic inflammation of a gallbladder mucosa and it is usually associated with gallstones [1]. However, the mechanisms leading to this pathology are not fully understood [2]. In light of recent studies, chronic inflammatory conditions are tightly related to tissue procoagulation state [3]. In this context, tissue factor (TF; CD142) transmembrane receptor and cofactor for clotting factor VII/VIIa have been reported to play a principal role in the initiation of inflammation-induced coagulation [4]. Accordingly, blocking TF activity inhibited inflammation-induced thrombin generation in the experimental model of bacteraemia [5]. In contrary, tissue factor pathway inhibitor (TFPI) provides anticoagulative and anti-inflammatory tissue activity by inhibiting the TF:FVIIa complex and factor Xa [6]. According to the abovementioned, the purpose of this study was to characterise TF and TFPI phenotype expression in relation to severity of inflammatory cell infiltration of gallbladder mucosa.

## 2. Patients and Methods

We prospectively studied the serial cryostat sections of the gallbladder specimens obtained from 54 consecutive patients (mean age, 57.3 ± 16.2 years; 10 males and 44 females) who had undergone cholecystectomy (due to symptomatic cholesterol gallstones) under the clinical diagnosis of chronic cholecystitis. The control group contains 16 calculosis-free gallbladder specimens obtained from patients (mean age, 53.7 ± 15.1 years; 5 males and 11 females) who underwent cholecystectomy due to the polyp/polyps as well as in cases of gallbladder injury. The blood samples were immediately chilled to 4°C, centrifuged, and analyzed immediately or frozen at −70°C until laboratory analysis. In addition, body mass index (BMI) (weight/height^2^; kg/m^2^) was used as an estimate of overall adiposity. For histology, a minimum five specimens per patient from the fundus of gallbladder were obtained. For immunohistology, all specimens were immediately fixed for 20 min in cold acetone (−20°C) and immersed in embedding medium (OCT Compound, Miles Inc.), and all of them were cut serially into 5 *μ*m thickness, air-dried at room temperature, and assayed. Frozen sections were incubated with murine monoclonal anti-human TF (clone TF9-10-H10 from American Diagnostica; the final dilution of 1 : 400) and anti-human TFPI (Abcam, ab66544; dilution 1 : 200). To suppress nonspecific staining due to endogenous alkaline phosphatase activity, 1% acetic acid (ChemPur) was used. The EnVision method (DAKO EnVision Kit/Alkaline Phosphatase detection system) was used according to the manufacturer's instructions. The bound primary antibody was detected using New Fuchsin Substrate System (DAKO A/S). The primary antibody was omitted from negative control slides. As a positive control, we used myocardial cryostat sections from heart. The sections were counterstained with Mayer's haematoxylin. Each specimen was evaluated qualitatively, semiquantitatively, (score index from 0 to 3+), and quantitatively. Semiquantitative score index was as follows: (0): no staining; (1+): weak focal staining; (2+): multifocal moderate staining; and (3+): diffuse strong staining. Cells positive for CD3 (clone T3-4B5) and CD68 (clone EBM11) were counted in all cryostat sections in at least 6 high power fields (HPF) per each biopsy under 400x magnification and averaged for each field using Nikon Eclipse 80i microscope with DS-F*i*1 digital camera and *NIS* Elements software form Nikon. All patients gave their informed consent. The protocol was approved by the institutional ethics committee.

## 3. Statistical Analysis

The baseline comparisons of the studied groups (cholecystitis versus control) were performed using the Mann-Whitney *U* test. To assess the relationship between quantitative data, the Spearman's rank-order coefficient was used, but the Kendall's tau rank-correlation coefficient test was used to assess the relationship between semi-quantitative data. Differences were considered statistically significant when  *P* < 0.05. The statistical analyses were performed using SPSS software package, v. 16.0.

## 4. Results

The clinical characteristics of the patients with chronic cholecystitis are listed in Table 1, but the results of immunoreactivity for TF and TFPI in the gallbladder mucous are summarized in Table 2.

**Figure 1:**
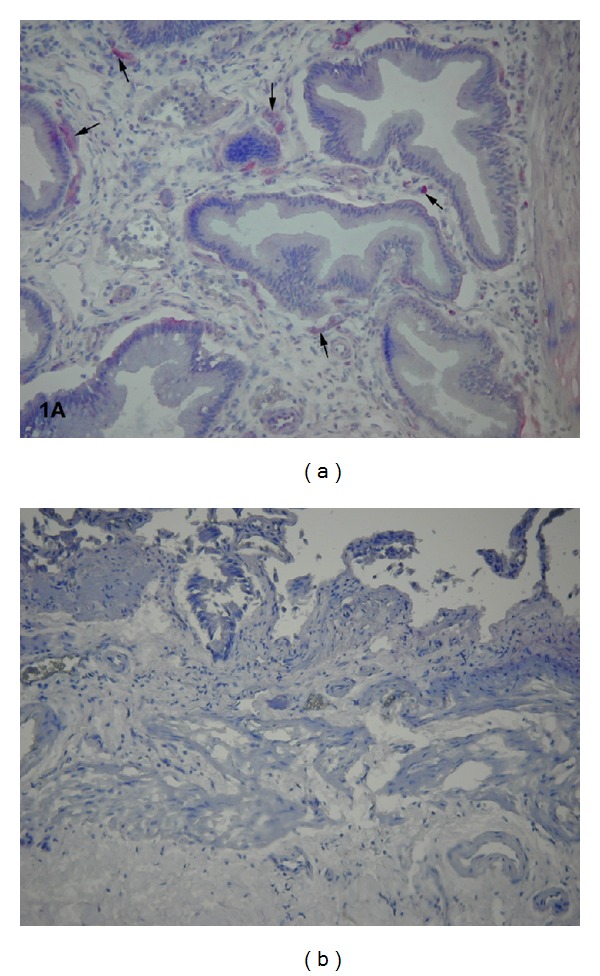
(a) Cryostat section from the cholecystitis group. Moderate to severe expression of the tissue factor on small microvessels and interstitial cells (arrows) (final magnification, ×150). (b) Cryostat section from the control group with lack of TF staining (final magnification, ×100).

The phenotype expression of the mucosal TF and TFPI differed significantly between the cholecystitis and the control group. Accordingly, moderate or strong TF expression was detected in the mucosal endothelial cells lining capillary vessel and in a few interstitial cells of the cholecystitis group (Figure 1(a)).

In the uninflamed mucosa of the control group, the endothelial and other interstitial cells were negative for TF (Figure 1(b); Table 2).

**Figure 2:**
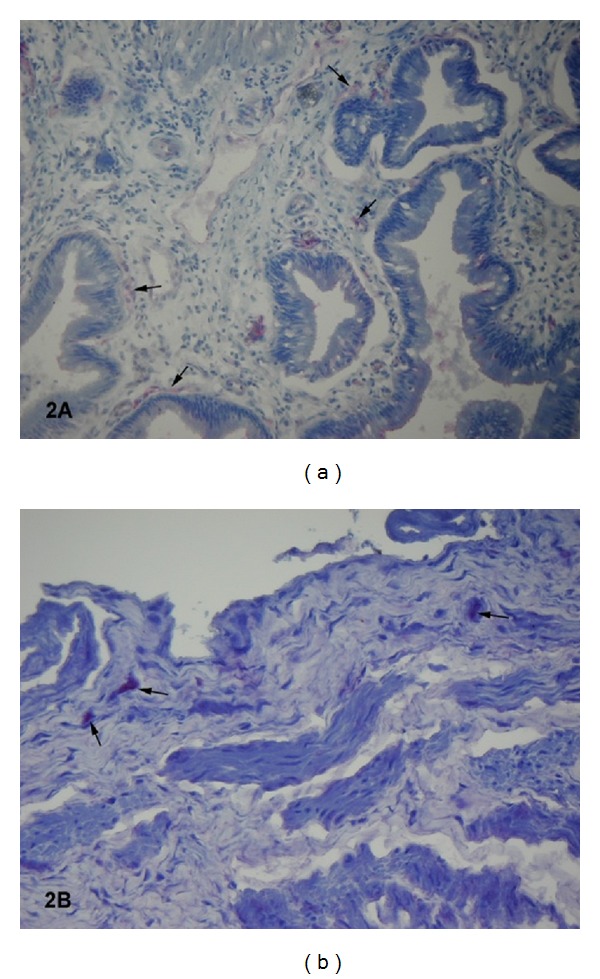
(a) Cryostat section from the cholecystitis group. Capillary endothelial cells presented weak tissue factor pathway inhibitor staining (arrows) (final magnification, ×150). (b) Cryostat section from the control group. Endothelial cells presented moderate staining for tissue factor pathway inhibitor (arrows) (final magnification, ×100).

The mucosal TFPI expression differed from the TF staining pattern. The most capillary endothelial cells in the cholecystitis group presented weak immunoreactivity for TFPI (Figure 2(a)).

A moderate expression for TFPI was only occasionally seen in the scattered capillary endothelial cells in such patients. Unlikely, in the control sections, the majority of endothelial cells presented moderate staining for TFPI (Figure 2(b); Table 2).

The mean number of CD3 positive lymphocytes in the cholecystitis group was 18.6 ± 12.2, but mean number of CD68 positive cells was 29.7 ± 13.9. In the control sections, it was 3.1 ± 1.9 and 8.8 ± 3.9, respectively (*P* < 0.001).

The expression of TF and TFPI showed no relation with clinics of the studied patients. In addition, there was no correlation between the severity of inflammatory cell infiltration of gallbladder mucosa and studied markers of tissue haemostasis.

## 5. Discussion

To the best of our knowledge, we for the first time demonstrated a procoagulant state in gallbladder tissue of the patients with clinically diagnosed chronic calculous cholecystitis. In addition, the current study revealed predominance of macrophages in cellular inflammatory infiltration in the examined sections of the patients with chronic cholecystitis. Unexpectedly, there was no relationship among tissue hemostatic markers studied, inflammatory infiltration severity, and clinical data in such cohort of patients.

It has been postulated that inflammatory mechanisms shift the haemostatic balance to favour activation of the coagulation [7]. It is known, that the main role in inflammation-induced coagulation is associated with TF [4]. The current study revealed upregulation of TF in the chronically inflamed gallbladder mucosa as compared to the control subjects. However, in the majority of the cholecystitis patients, increased TF expression was not associated with upregulation of TFPI in the studied sections.

Tissue factor pathway inhibitor is a principal inhibitor of TF induced coagulation by inhibition of both factor Xa and a complex of TF and factor VIIa [8]. This inhibitor is synthesised by the endothelial cells and in majority is associated with the vessel wall [9]. Both lack of upregulation and downregulation of TFPI as compared with the healthy subjects found in the current study may be most likely due to cleavage of TFPI by proteases of inflammatory cells [10]. It is worth emphasising that, as it was previously reported, the lack of TFPI upregulation may be important contributing factor responsible for procoagulation state in acute and chronic inflammatory conditions [11, 12].

This study failed to demonstrate any relationship between tissue markers of hemostasis studied and cell inflammatory infiltration. It may be partially explained by time-dependent different histological patterns of the inflammatory infiltration in chronically inflamed gallbladder mucous [13, 14].

In conclusion, this study revealed the presence of upregulation of TF expression accompanied by TFPI downregulation in the chronic calculous cholecystitis irrespective of the disease severity. It may suggest that hemostasis disturbances are engaged in the etiopathogenesis of the cholecystitis. However, the clinical relevance of these findings needs further elucidation.

## Figures and Tables

**Table 1:** Clinical and demographic data.

	Cholecystitis (*n* = 54)
Age, y	57.3 ± 16.2
Sex, male/female	10/44
Hypertension, *n* (%)	31 (57.4)
Diabetes mellitus, *n* (%)	5 (9.3)
Coronary artery disease, *n* (%)	15 (27.8)
BMI*, kg/m^2^	26.1 ± 4.7
Fibrinogen, g/L	5.5 ± 1.4
Bilirubin, U/L, median (1st–3rd quartiles)	11.7 (8.3–21.1)
ALT*, U/L, median (1st–3rd quartiles)	46 (28–93.5)
AST*, U/L, median (1st–3rd quartiles)	44 (28–61)
GGTP*, U/L, median (1st–3rd quartiles)	32 (18–56)
ALP*, U/L	77.7 ± 24.7

*BMI: body mass index; *ALT: alanine aminotransferase; *AST: aspartate aminotransferase; *GGTP: *γ*-glutamyltransferase; *ALP: alkaline phosphatase.

**Table 2:** Number (percentage) of patients studied within each of TF and TFPI scores and mean number of CD68 and CD3 positive cells.

Haemostasis and cell markers studied	Score	Control *n* = 16	Cholecystitis *n* = 54	P*
TF, *n* (%)	≤1+	15 (93.7)	15 (27.8)	<.001
	2+	1 (6.3)	15 (27.8)	
	3+	0 (0)	24 (44.4)	

TFPI, *n* (%)	≤1+	2 (12.5)	31 (57.4)	<.001
	2+	13 (81.2)	15 (27.8)	
	3+	1 (6.3)	8 (14.8)	

CD3+, mean ± SD	—	3.1 ± 1.9	18.6 ± 12.2	<.001

CD68, mean ± SD	—	8.8 ± 3.9	29.7 ± 13.9	<.001

*Cholecystitis group versus control.
